# A National Pension Fund for Hospital Officials and Nurses

**Published:** 1887-06-11

**Authors:** 


### A National Pension Fund for Hospital Officials and Nurses.

THE VIEWS OF OFFICIALS, SISTERS, NURSES, AND OTHERS.

### From the Matron of Lancaster Infirmary.

I beg to send you my name and that of my trained nurse as subscribers to the National Pension Fund for Nurses. My two probationers are very pleased at the thought of being able to make some provision for sickness or old age, and will join when they obtain their certificates. Has a pamphlet been printed setting forth the nature of the scheme? If so, kindly send me three, as I wish to send two to nurses who have left me, and one to a district nurse here who may not hear of it. I sincerely hope that the Fund may be established this year. Hospital officials and nurses ought to be very grateful to the Editor of THE HOSPITAL for so kindly advocating the scheme, which if successful, will be a great boon to all. With heartiest thanks to the Editor.

### By a Trained Nurse willing to Subscribe.

"A TRAINED NURSE, Aberfeldy" (see THE HOSPITAL, No. 33, p. 108), writes:—"I expected an answer in the columns of THE HOSPITAL to enlighten others who are in the same state of ignorance as myself concerning the Trained Nurses' Annuity Fund—when established, where situated, what amount of contribution entitles a member to the benefits it allows, its terms, and manager's name? Perhaps, if the existence of this Fund were more widely known, or if necessary its formula altered to take in the idea of the National Pension Fund, the very laudable object might be achieved without the preliminary expenses being very heavy, and the working expenses very little more than at present. I shall be glad to contribute. The reference to the Trained Nurses' Annuity Fund was made in THE HOSPITAL, April 2nd."

